# Relationship between cancer stem cell-related SNPs and survival outcomes in patients with primary lung cancer

**DOI:** 10.1186/s12957-023-03064-z

**Published:** 2023-08-11

**Authors:** Xinying Xu, Yuhang Liu, Huiyi Hu, Jinshen Wang, Yuxin Cai, Jun Xie, Mingqiang Kang, Fei He

**Affiliations:** 1 Department of Epidemiology and Health Statistics, School of Public Health, Fujian Medical University, Fuzhou, China; 2 Department of Labor Health, School of Public Health, China Medical University, Shenyang, China; 3 Department of Venereal Disease Prevention, Dermatology Hospital, Southern Medical University, Guangzhou, China; 4 Department of Health Toxicology, School of Public Health, Xiamen University, Xiamen, China; 5 Sanming Dermatology Hospital, Sanming, China; 6 Department of Thoracic Surgery, Fujian Medical University Union Hospital, Fuzhou, China

**Keywords:** Lung cancer stem cells, Single-nucleotide polymorphism, Prognosis, Gene

## Abstract

**Background:**

Cancer stem cells may be the source of cancer-causing mutant cells and are closely related to the prognosis of cancer. Our study aimed to investigate the potential association between single-nucleotide polymorphisms (SNPs) of cancer stem cell-related genes and the prognosis of lung cancer patients.

**Methods:**

The SNP loci were genotyped by matrix-assisted laser desorption ionization time of flight mass spectrometry (MALDI-TOF–MS), and the overall survival of subjects was analyzed by log-rank test after stratifying and adjusting their demographic data, clinical data, and genotypes. The correlation between survival time and quality of life of lung cancer under codominant, dominant, recessive, and additive genetic models was analyzed by the Cox regression model. The association between SNP polymorphism and the prognosis of lung cancer was analyzed by Stata16.0 software, and their heterogeneity was tested. Interaction analysis was performed using R software (version 4.2.0).

**Results:**

Stratified analysis unveiled that rs3740535 had recessive AA genotype and additive GG genotype; Rs3130932 dominant GT + GG genotype, additive TT genotype; Rs13409 additive TT genotype; Rs6815391 recessive CC genotype and additional TT genotype were associated with increased risk of lung cancer death. Rs3130932 recessive GG genotype was associated with a reduced risk of lung cancer death.

**Conclusion:**

Rs3740535, rs3130932, rs13409, and rs6815391 are associated with the overall survival of lung cancer patients and may be valuable for the prognosis of lung cancer patients.

**Supplementary Information:**

The online version contains supplementary material available at 10.1186/s12957-023-03064-z.

## Introduction

Primary lung cancer is one of the most common malignant tumors in the world. The incidence and mortality of lung cancer are increasing in the world. The mortality rate of lung cancer reaches 1–5% annually, especially in China and other developing countries [1, 2]. Lung cancer is the number one cause of death in the world. In 2020, lung cancer deaths were estimated at 1.8 million worldwide [3]. China now has the largest number of lung cancer patients in the world, and lung cancer is one of the most common cancers and a leading cause of the cancer-related deaths in China [4]. There were 828,000 new cases of lung cancer reported in China in 2016, including 555,000 men and 278,000 women, accounting for 20.4% of all malignancies, according to the China Tumor Registration Center [5]. Understanding the etiology, prevention, diagnosis, and treatment of lung cancer is critical for management of lung cancer patients in the world. Epidemiological studies have shown that the development of lung cancer is attributed to the combined action of environmental factors and genetic factors. Although tobacco exposure causes more than 80% of lung cancer [6], under the same tobacco exposure, only less than 20% of smokers develop lung cancer [7]. These suggest that individuals have varying genetic susceptibility to lung cancer. Smoking is a major risk factor for the development of lung cancer, especially among men. Information from the China Health and Nutrition Survey has shown that the smoking age of the Chinese population is decreasing yearly [8]. Genetic predisposition to smoking is associated with increased rates of lung adenocarcinoma and lung squamous cell carcinoma [9].

Cancer stem cells (CSCs) have the capacity to self-renew and differentiate into different types of cells and may be responsible for tumor formation, maintenance, and metastasis [10, 11]. There is growing evidence that stem cells may be the source of mutated cells that drive cancer development. A previous study has revealed that 25 SNPs in the stem cell-related genes are significantly associated with the development of lung cancer the dominant genes include *RAN* rs14035, *TP53INP1* rs7760, *TP53INP1* rs896849, *EPCAM* rs1126497, *HEY1* rs1046472, *HEY2* rs3734637, *OCT4* rs13409, and *WNT2* rs3729629 [12]. It is well known that the octamer-binding factor 4 (*OCT4*) is a critical transcription factor for the stemness of cancer stem cells and regulates the occurrence, development, and metastasis of lung cancer [13]. Furthermore, *OCT4* can bind to the promoter or enhancer region of lncRNAs to induce their expression [14]. *OCT4* knockdown can significantly increase the apoptosis of lung cancer cells, inhibit tumor growth, and prolong the survival of mice, but it can not cure lung cancer [15, 16]. Transcription factors in embryonic stem cells interact to form networks that collectively regulate the fate of embryonic stem cells. *OCT4*, *NANOG*, *KLF4*, *C-MYC*, and *SOX2* are the major transcription factors to regulate stem cell pluripotency. A recent study indicates that the Rac exchange factor 1 (*REX1*) may replace *KLF4* to maintain multiple capabilities and reprogramming of cancer stem cells [17]. Moreover, the *REX1* promoter contains the binding sites of multiple core transcription factors, but it has bidirectional regulation on the *OCT4* gene and plays an effect on the pluripotency of cancer stem cells. T lymphoma invasion and metastasis induction gene 1 (*RAC GTPase*) is implicated in the downstream regulation of the *OCT4* gene. The interaction between Tiam1 and C-terminal binding protein 2 (*CTBP2*) promotes the proto-oncogenic function of *CTBP2* and leads to cancer cell migration [18].

Based on prognostic data from 61 countries worldwide, the age-standardized 5-year net survival rates of lung cancer range from 10 to 20% [19]. Although the diagnosis and treatment of lung cancer in China have made a great advance in the past decades, the current age-standardized 5-year survival rate remains less than 20.0% [20]. Many factors can affect the prognosis of lung cancer, including tumor histopathological type, TNM stage, treatment, gender, and age. However, clinical data reveal that the same group of lung cancer patients still have different prognoses. Hence, genetic factors are important for the prognosis of lung cancer. The discovery of effective biomarkers is of great significance for the prognosis of lung cancer. Therefore, this study intended to explore the potential association between SNPs of upstream *REX1* of *OCT4*, downstream *CTBP2* of *OCT4* and *OCT4*, and the prognosis of lung cancer. Our findings may help in evaluating the prognosis of lung cancer patients and guide the clinical development of more targeted treatment.

## Material and methods

### Object of study

The subjects were collected from the Department of Thoracic Surgery of the First Affiliated Hospital of Fujian Medical University, Union Hospital of Fujian Medical University and Fuzhou General Hospital of Nanjing Military Command. Inclusion criteria were as follows: (1) new cases of primary lung cancer confirmed by bronchoscopy or surgical histopathology, (2) the time of diagnosis was from January 2006 to December 2012, and (3) have lived locally in Fujian for more than 10 years. The exclusion criteria were patients with pathologically diagnosed lung inflammation, benign lesions, secondary lung cancer, and patients in critical condition who could not clearly answer the questions. This study was approved by the Ethics Committee of Fujian Medical University, and all subjects signed informed consent before the investigation began.

## Methods

### Specimen collection and processing

Peripheral venous blood samples (5 ml each) were collected from individual patients with primary lung cancer before any antitumor treatment and centrifuged for 10 min. The anticoagulant blood samples were divided into the whole blood, plasma, and blood cell samples and stored in a − 80 ℃ freezer. Genomic DNA was extracted from individual whole blood samples using the AxyPrep Blood Genomic DNA Miniprep Kit (Axygen Biotechnology, Tewksbury, MA, USA). The quality and quantification of each genomic DNA sample was analyzed by a NanoDropTM ND-1000 ultraviolet spectrophotometer.

### SNP site selection

The *OCT4*, *REX1*, and *CTBP2-*related inflammatory signaling pathways to lung cancer were evaluated using the KEGG (Kyoto Encyclopedia of Genes and Genomes) database (http://www.genome.jp/kegg/). The key node genes were searched, and their sequences were identified using the NCBI gene functional annotation database (http://www.ncbi.nlm.nih.gov/gene/). The criteria for the selection of functional SNP sites included (1) being located in the coding region and might affect protein-coding and properties; (2) located in the binding region of transcription factors and might affect the transcriptional activity and the gene expression; (3) located in the shearing site region, and might affect the selective shearing function of mRNA, and the protein function; (4) located in the 3′UTR region, namely the microRNA binding region, and might affect the binding activity of microRNA, and the process of post-transcriptional modification of mRNA. Accordingly, four functional SNP sites were selected and included rs13409, rs6815391, rs3740535, and rs3130932 in *OCT4*, *REX1*, and *CTBP2* genes for subsequent analysis.

### SNP genotyping

The SNP loci were genotyped by matrix-assisted laser desorption ionization time of flight mass spectrometry (MALDI-TOF–MS) (Sequenom, USA), and 5% of all samples were randomly selected for repeated detection by internal quality control to ensure the accuracy of genotyping.

#### The principle of MALDI-TOF–MS detection

Because the interesting PCR fragments of the end products had different charge/mass ratios, when they were labeled with analyte ions they had varying flight times, leading to the different arrival times in the detector to generate sequential current signals. Accordingly, these different masses of PCR fragments were distinguished and identified by the MALDI-TOF–MS. The whole detection process included two steps. Firstly, PCR amplification primers (same as conventional sequencing amplification primers) were designed, gene PCR fragments were purified, and the specific probe primers for mutation sites were designed. The mixed products of mutant/wild-type loci gene fragments were obtained by the iPlex single base extension experiment. Finally, these different fragments were identified by mass spectrometry. Sample DNA extraction, gene amplification reaction system, and conditions were strictly performed, according to the operating instructions of Sequenom complete PCR Reagent Set. The probe primer extension reactions were strictly performed, in accordance with the Sequenom iPLEx Gold ReagentKjc operation instructions. Assay design 2.0 (Sequenom) was used to design the probe primers according to the NCBI reference sequence. The test results were analyzed by Typer 4.0 (Sequmon) software, supplemented by manual correction of the secondary peak map.

#### Experimental procedure

1. PCR amplification: the total volume of 5.0 µl reaction solution consisted of 1.0 µl DNA sample, 1.8 µl primary water, 0.5 µl 10 × PCR buffer, 0.4 µl MgCl_2_, 0.1 µl dNTP, 0.2 µl PCR enzyme, and 1.0 µl PCR primer; Reactions were performed for predenaturation at 94℃ for 4 min and subjected to 45 cycles of denaturation at 94℃ for 20 s, annealing at 56℃ for 30 s, and extension at 72℃ for 1 min, followed by the final extension at 72℃ for 3 min. The PCR products were stored at 4℃.
2. Purification of PCR products: The PCR products were first digested with shrimp alkaline phosphatase (SAP) (using 0.17 μl of 10 × SAP buffer and 0.3 μl SAP enzyme) at 37℃ for 40 min and at 85℃ for 5 min and cooled down at 4℃ to remove the residual dNTP.
3. Single-base extension reaction: The iPlex reactions were performed in a total volume of xx μl including 0.2 μl of 5 × iPlex buffer, 0.6 μl of primary water, 0.2 μl of iPlex terminator, 0.94 μl of primers, and 0.041 μl of iPlex enzyme at 94℃ for 30 s and subjected to 40 cycles of denaturation at 94℃ for 5 s, 52℃ for 5 s, and 80℃ for 1 min, followed by 5 cycles of 52℃ for 5 s and 80℃ for 1 min and finally extended at 72℃ for 3 min. The products were stored at 4℃.
4. MALDI-TOF–MS analysis of samples: The iPlex reaction products were transferred using a Nanodispenser (Sequenom) from the microplates into SpectroCHIP (Sequenom), and the data will be acquired using the MassARRAY analyzer and analyzed by MALDI-TOF–MS. Genotyping results were analyzed by MassArrayTyper 4.0 software.

### Data collection and follow-up

Individual cases were collected with their data, including gender, age, pathological type, stage, surgery, tumor sizes, chemotherapy, radiotherapy, metastasis status and locations, pleural metastasis, lymphatic metastasis, hematogenous metastasis, body mass index (BMI), other lung diseases, and family history of tumors. The enrolled cases were followed up for tumor recurrence, metastasis, treatment, and survival status (death, cause of death, date of death) every 6 months up to December 1, 2020.

### Definition of variables

BMI = weight (kg)/[height (m)]^2^. According to the Chinese standard [21], 18.5 kg/m^2^ ≤ BMI < 24.0 kg/m^2^ is considered normal, and BMI < 18.5 kg/m^2^ is considered underweight while BMI ≥ 24.0 kg/m^2^ is considered overweight or obese. Smoking refers to the cumulative smoking of more than 100 cigarettes in a lifetime. A family history of cancer refers to a history of malignancy in relatives, such as parents, siblings, children, grandparents, maternal grandparents, uncles, and aunts.

### Statistical treatment

Statistical analyses were performed using SPSS 25.0 software. The difference in the survival periods among the groups was analyzed by log-rank test. The correlation between survival time and quality of life of lung cancer under codominant, dominant, recessive, and additive genetic models was analyzed using the Cox regression model. The association of SNP polymorphism with and prognosis of lung cancer was analyzed by Stata16.0 software, and the heterogeneity of the data was tested. The potential interactions between variates were tested using R software (version 4.2.0). A *p* value of < 0.05 was considered statistically significant.

## Results

### General information of the research subjects

There were 1059 lung cancer patients included in this study, and the distribution of general demographic and clinical data of those patients is shown in Table 1. The median survival time of all subjects was 24.33 months, the minimum age was 23 years, the maximum age was 86 years, and the mean age was 59.0 ± 10.7 years. There were 774 males (73.10%), 285 females (26.90%), and 671 smokers (63.90%). The pathological types were mainly adenocarcinoma, squamous cell carcinoma, and small cell lung cancer. 572 cases (54.00%), 297 cases (28.00%), and 85 cases (8.00%), respectively. There were 457 patients (43.20%) in early stage, 602 patients (56.80%) in advanced stage, 552 patients (52.10%) in surgical treatment, 749 patients (70.70%) in chemotherapy, and 208 patients (19.60%) in radiotherapy. By October 11, 2020, 1045 cases were completely followed-up, with a follow-up rate of 98.7%, 14 cases (1.3%) missing office visits due to personal reasons, and 227 cases remained survival (21.4%), while 818 cases died (77.2%).

**Table 1:** Basic information distribution table of lung cancer patients

Variable	Variable	Number of cases (*n* = 1059)	Constituent ratio (%)
Sex	Male	774	73.10
	Female	285	26.90
Age	< 60	527	49.80
	≥ 60	532	50.20
Smoking	Yes	671	63.90
	No	379	36.10
Pathological type	Adenocarcinoma	572	54.00
	Squamous carcinoma	297	28.00
	Others	190	18.00
Clinical stages	Early stage	457	43.20
	Later stage	602	56.80
Operation	Yes	552	52.10
	No	507	47.90
Chemotherapy	Yes	749	70.70
	No	310	29.30
Radiotherapy	Yes	208	19.60
	No	851	80.40

### Association of gene polymorphism and the overall survival of lung cancer patients

Cox proportional risk model was used to explore the relationship between SNPs and overall survival of lung cancer. Considering that multiple factors may affect the prognosis of lung cancer patients, the SNPs and overall survival time were analyzed by univariate analysis and multivariate analysis. The overall survival of lung cancer with different SNP genotypes is shown in Table 2. Log-rank test indicated that there was no significant difference in overall survival time between different SNPs genotypes (*P* > 0.05).

**Table 2:** The association of SNPs with the overall survival of lung cancer patients

Genetic locus	Genotype	Lung cancer		
		Deaths/total number	Log rank *P*	*αHR* (95%*CI*)^*^
rs13409				
Co-dominance	CC	396/507	0.965	1
	CT	292/379		0.948(0.815–1.104)
	TT	87/116		0.919(0.728–1.161)
Dominance	CC	396/507	0.831	1
	CT + TT	379/495		0.941(0.817–1.084)
Recessiveness	CC + CT	688/886	0.819	1
	TT	87/116		0.940(0.752–1.177)
Additivity			0.792	0.928(0.735–1.172)
rs6815391				
Co-dominance	TT	365/473	0.541	1
	CT	333/433		1.075(0.926–1.249)
	CC	78/99		1.180(0.924–1.508)
Dominance	TT	365/473	0.347	1
	CC + CT	411/532		1.094(0.949–1.260)
Recessiveness	CT + TT	698/906	0.395	1
	CC	78/99		1.141(0.902–1.442)
Additivity			0.292	1.169(0.915–1.494)
rs3740535				
Co-dominance	GG	467/587	0.667	1
	AG	275/366		0.948(0.816–1.101)
	AA	58/74		0.889(0.676–1.169)
Dominance	GG	467/587	0.371	1
	AA + AG	333/440		0.937(0.814–1.079)
Recessiveness	AG + GG	742/953	0.846	1
	AA	58/74		0.907(0.694–1.186)
Additivity			0.721	0.895(0.680–1.177)
rs3130932				
Co-dominance	TT	350/442	0.497	1
	GT	101/451		0.959(0.827–1.113)
	GG	92/129		0.804(0.638–1.013)
Dominance	TT	350/442	0.299	1
	GG + GT	440/580		0.923(0.802–1.062)
Recessiveness	GT + TT	698/893	0.382	1
	GG	92/129		0.821(0.660–1.022)
Additivity			0.285	0.810(0.643–1.022)

Note: *Adjusting factors were age, sex, pathological type, presence or absence of treatment, and clinical stages

We further stratified patients, based on their age, gender, pathological type, with or without treatment, and clinical stages of adjustment factors, and used the Cox proportional hazards models to analyze the overall survival of patients with different SNPs with various genetic models. The hazard ratio (HR) and 95% confidence interval (CI) were calculated in Table 2. There was no single-tested SNP regardless of genetic models that was significantly associated with the overall survival of this population of lung cancer patients.

### Stratification analysis of SNPs polymorphism and prognosis of lung cancer

**Fig. 1:**
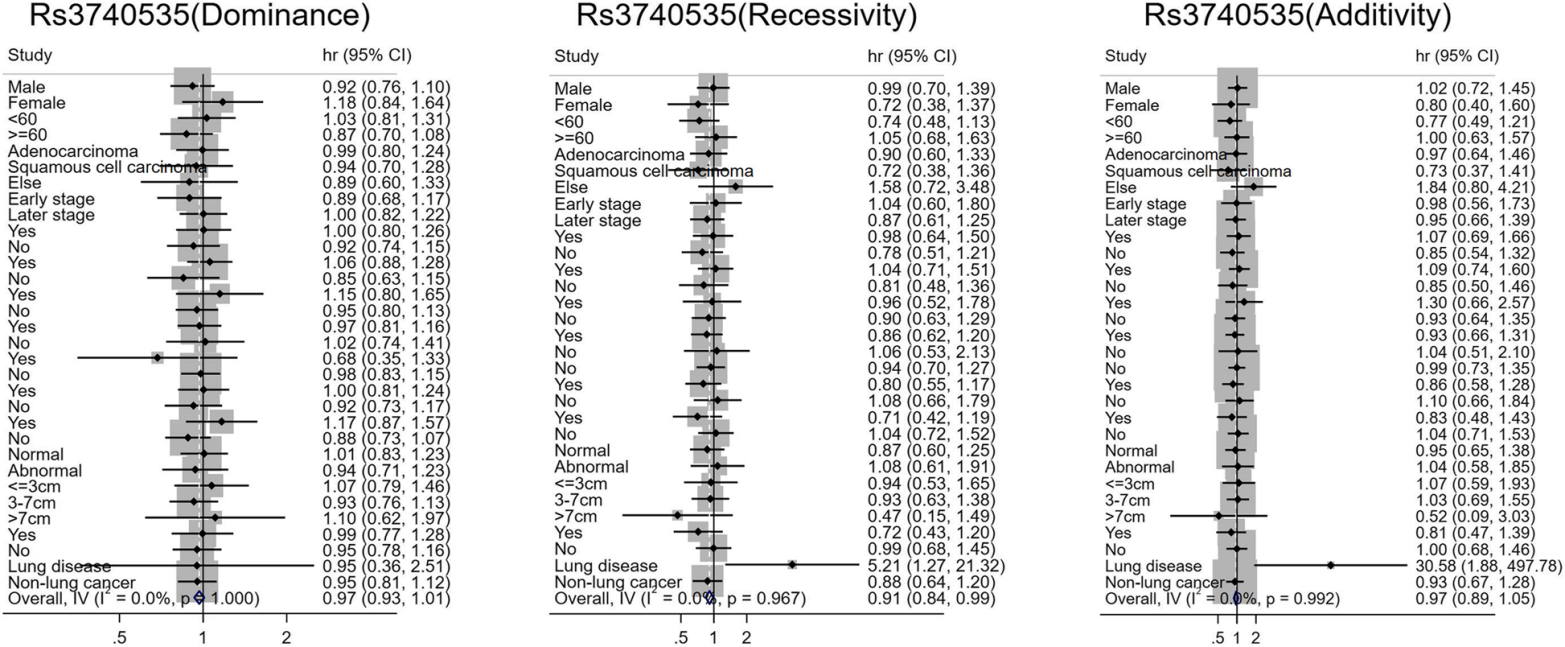
Stratified analysis of rs3740535 and lung cancer prognosis

**Fig. 2:**
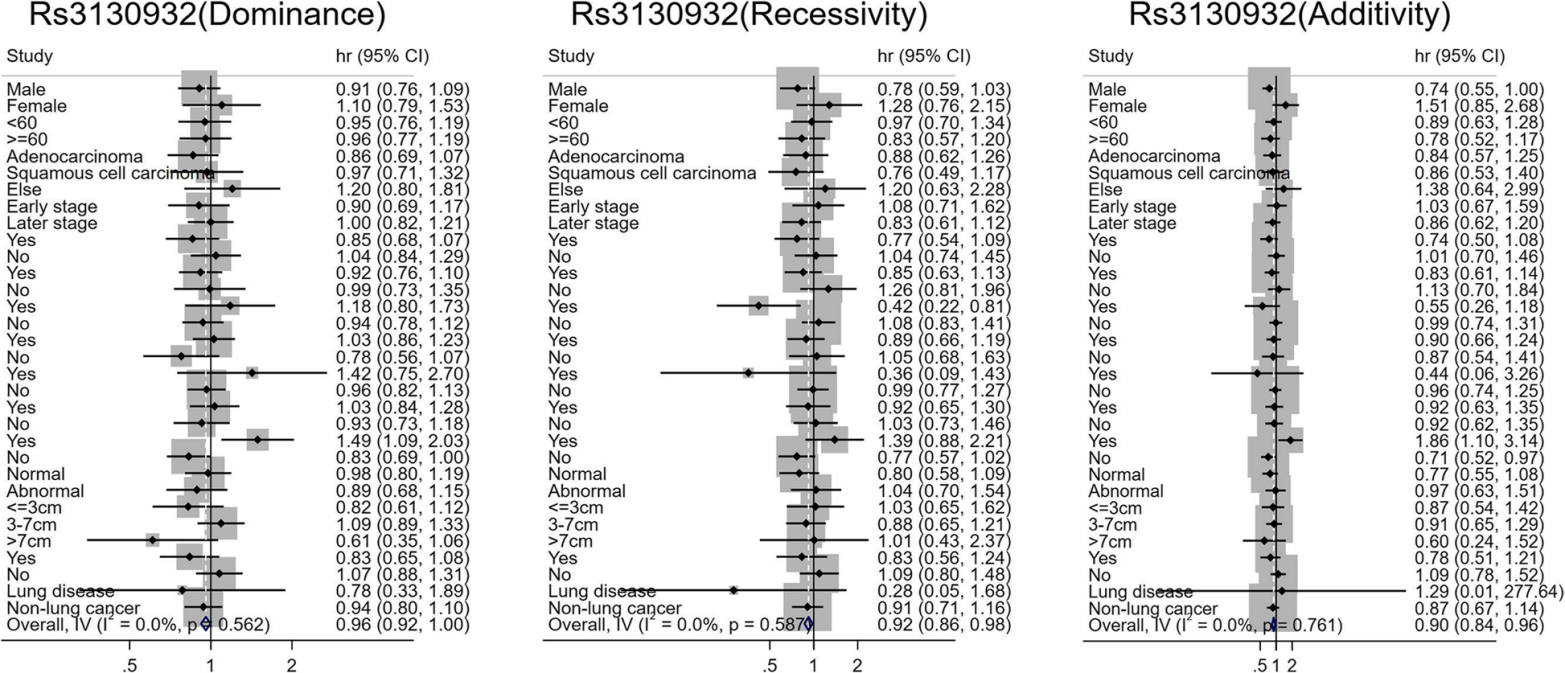
Stratified analysis of rs3130932 and prognosis of lung cancer

**Fig. 3:**
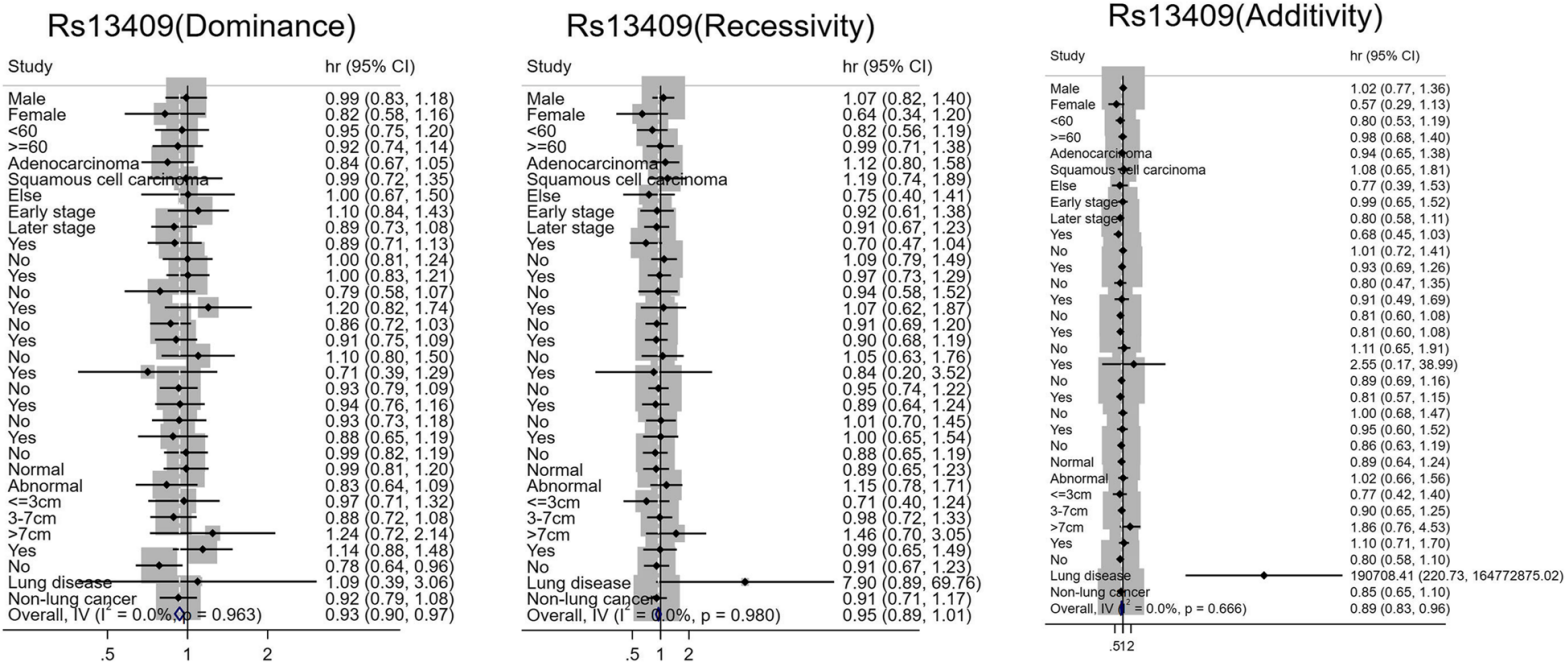
Stratified analysis of rs13409 and prognosis of lung cancer

**Fig. 4:**
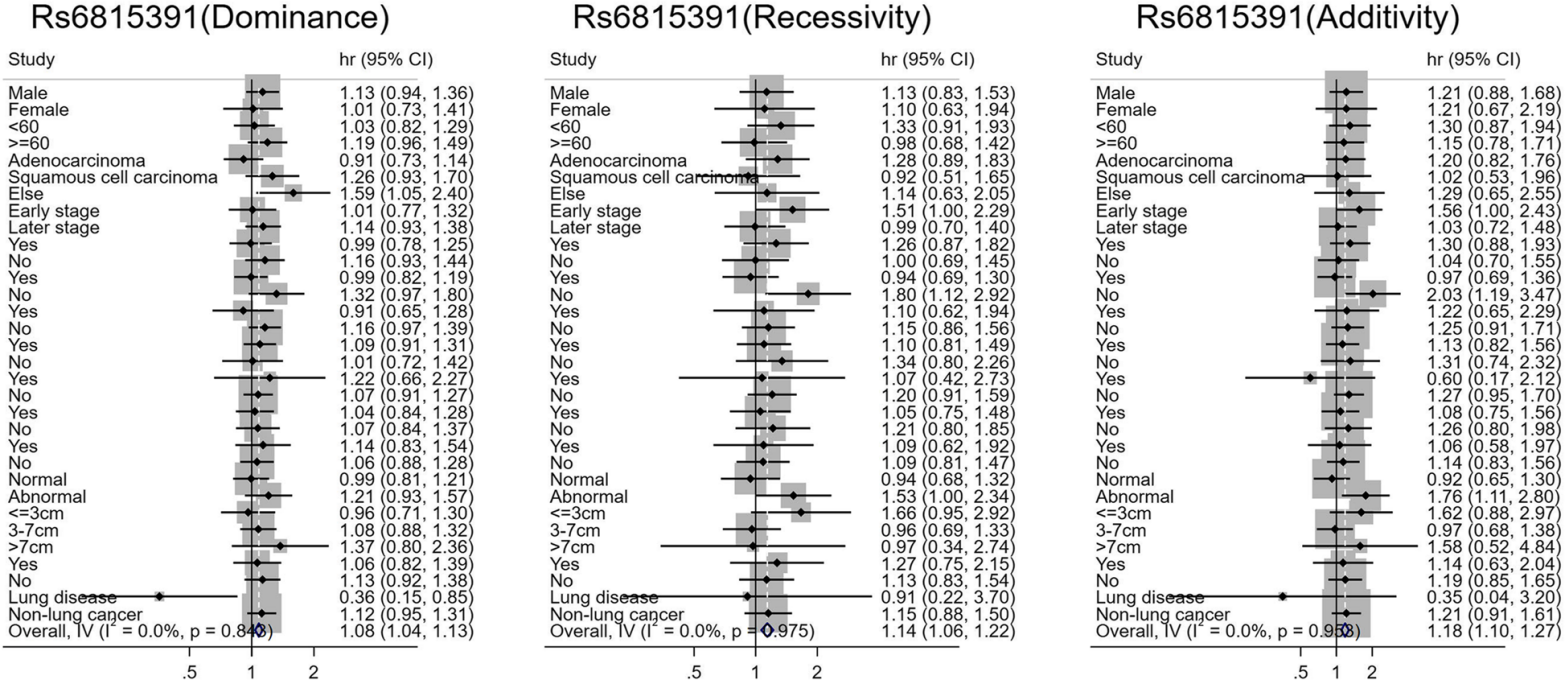
Stratified analysis of rs6815391 and prognosis of lung cancer

SNPs were divided into dominant, recessive, and additive genetic models. To explore the effects of SNP polymorphism changes on patients with different demographic and clinical characteristics, those patients were stratified, based on the collected clinical data in Figs. 1, 2, 3, and 4. Interestingly, the risk effect of recessive and additional rs3740535 on prognosis was significant in patients with a family history of lung cancer. Among recessive genotypes, the risk of death in patients with rs3740535 AA genotype was 5.210 times higher than that in patients with AG + GG genotype (95% *CI* 1.273–21.324). Among the additional genotypes, the risk of death in patients with rs3740535 GG genotype was 30.583 times higher than that in patients with AA genotype (95% *CI* 1.879–497.832). Heterogeneity test exhibited that there was significant heterogeneity of this risk effect in both recessive and additive genotypes of rs3740535 with or without a family history of lung cancer (*P*_*heterogeneity*_ < 0.05) (Fig. 1).

Rs3130932 has both dominant and additive effects on the prognosis of patients with hematogenous metastasis. In the dominant genotypes, patients with the rs3130932 GT + GG genotype had 1.492 times higher risk of death than patients with the TT genotype (95% *CI* 1.094–2.034). Among the additional genotypes, the risk of death in patients with blood type transfer was 1.856 times higher in patients with TT genotype than in patients with the GG genotype (95% *CI* 1.097–3.143). Heterogeneity test indicated that the risk effects of rs3130932 had significant heterogeneity between the dominant and additive genotypes with or without subgroups (all *P*_*heterogeneity*_ < 0.05). Rs3130932 had a recessive protective effect on the prognosis of patients receiving radiotherapy. Patients with the rs3130932 GG genotype had a significantly lower risk of lung cancer-related death than patients with the GT + TT genotype (*HR* = 0.420, 95% *CI* 0.217–0.814). Heterogeneity test revealed that this protective effect was significantly different between radiotherapy and non-radiotherapy subgroups (*P*_*heterogeneity*_ = 0.009, Fig. 2).

The risk effect of rs13409 on the prognosis of lung cancer was significant in patients with a family history of lung cancer. The risk of death in patients with the rs13409 TT genotype was 190,708.408 times higher than in patients with the CC genotype (95% *CI* 220.699–164,793,486.900). Heterogeneity test exhibited that patients with a family history of lung cancer and carrying the rs13409 additional TT genotype had a significantly higher risk of death than patients without a family history of lung cancer (*P*_*heterogeneity*_ < 0.01, Fig. 3).

In patients with pathological types other than adenocarcinoma and squamous cell carcinoma (*HR* = 1.590, 95% *CI* 1.053–2.401), rs6815391 dominant CT + CC genotype carriers were associated with an increased risk of death, and this risk effect was significantly different between subgroups of pathological types (*P*_*heterogeneity*_ = 0.037). Patients with a family history of lung cancer (*HR* = 0.358, 95% *CI* 0.150–0.852) had a significantly reduced risk of death, and this protective effect was significantly different between the subgroups with and without a family history of lung cancer (*P*_*heterogeneity*_ = 0.012). The recessive risk effect of rs6815391 on prognosis was more significant in patients who did not receive chemotherapy. The risk of death was 1.805 times higher in patients with CC genotype than in patients with the CT + TT genotype (95% *CI* 1.115–2.922). Heterogeneity test found that this risk effect was significantly different between subgroups with and without chemotherapy (*P*_*heterogeneity*_ = 0.027). In patients without chemotherapy (*HR* = 2.027, 95% *CI* 1.186–3.466) and abnormal BMI (*HR* = 1.763, 95%*CI* 1.110–2.800), rs6815391 patients with additional TT genotype had a higher risk of death than those with CC genotype. Heterogeneity test revealed significant heterogeneity between with and without chemotherapy subgroups (*P*_*heterogeneity*_ = 0.022) and statistically significant heterogeneity between normal and abnormal BMI subgroups (*P*_*heterogeneity*_ = 0.027, Fig. 4).

Based on the stratified results, we further analyzed the interaction between different genotypes and factors with heterogeneity among sublayers of clinical variables (Table 3, Additional file 1 tables 4 and 5) after adjusting for gender, age, initial diagnosis, pleural metastasis, lymphatic metastasis, hematologic metastasis, BMI, presence or absence of treatment, presence or absence of surgery, maximum tumor size, presence or absence of pulmonary disease, education, smoking history, clinical stages, and other variables.

**Table 3:** Analysis of interaction between rs3740535 genotypes and family history of cancer

Implicit model			Additive model		
Genotype	Family history of cancer	*αHR* (95%*CI*)^*^	Genotype	Family history of cancer	*αHR* (95%*CI*)^*^
AG + GG	No	1	AA	No	1
AG + GG	Other tumors	0.969(0.761–1.234)	AA	Other tumors	0.906(0.678–1.210)
AG + GG	Lung cancer	1.129(0.810–1.572)	AA	Lung cancer	1.179(0.752–1.847)
AA	No	0.949(0.669–1.346)	GG	No	1.009(0.706–1.443)
AA	Other tumors	0.677(0.347–1.321)	GG	Other tumors	0.704(0.358–1.385)
AA	Lung cancer	2.047(0.744–5.637)	GG	Lung cancer	2.130(0.758–5.981)
Multiplication interaction		1.035(0.912–1.175)	Multiplication interaction		1.019(0.873–1.190)
Relative excess risk (RERI)		0.125(− 0.434–0.685)	Relative excess risk (RERI)		0.121(− 0.434–0.675)
Attributable risk percent (ARP)		0.203(− 0.594–1.001)	Attributable risk percent (ARP)		0.207(− 0.692–1.106)
Synergic index (S)		0.754(0.172–3.307)	Synergic index (S)		0.775(0.205–2.931)

Note: * is adjusted for gender, age, first diagnosed metastasis, pleural metastasis, lymphatic metastasis, hematologic metastasis, BMI, whether there is treatment, whether there is surgery, a maximum diameter of tumor, whether there is a lung disease, education background, smoking history, and clinical stages (when adjusting factors are included, the corresponding combined items are not included in the adjustment)

There was no statistically significant effect of rs3740535 recessive and additive models on the combined effect, multiplicative interaction, and additive interaction of rs3740535, and family history of tumor (Table 3).

In the dominant model, patients with rs3130932 GT + GG genotype had 1.362 times higher risk of death than patients with rs3130932 TT genotype with hematoascular metastasis (95% *CI* 1.021–1.818). In the recessive model, the risk of death of lung cancer patients carrying rs3130932 GG genotype and receiving radiotherapy was 0.536 times that of lung cancer patients carrying rs3130932 GT + TT genotype and receiving radiotherapy (95% *CI* 0.298–0.962). Rs3130932 recessive had a significant positive multiplicity that interacted with radiotherapy. Patients with the rs3130932 GG genotype had 0.650 lower times risk of death than patients with the rs3130932 GT + TT genotype (95% *CI* 0.464–0.911). In the additive model, rs3130932 had no significant combined effect with blood metastasis and gender, but rs3130932 had significant positive multiplying interaction with gender. Rs3130932 polymorphism changes and blood metastasis in the three models displayed significant negative multiplicative interaction, but no significant additive interaction (Table 4, Additional file 1).

In the dominant model of rs6815391, there was no significant association between rs6815391 and a family history of tumors. Patients with rs6815391 TT genotype and pathological non-adenocarcinoma and squamous cell carcinoma types had a 0.707 times lower risk of death than patients with rs6815391 TT genotype and pathological adenocarcinoma type (95% *CI* 0.512–0.978). Rs6815391 dominance had no significant multiplication and addition interaction with tumor family history and pathological type. In the recessive model, the risk of death in patients without chemotherapy carried a rs6815391 CC genotype was 1.859 times higher than that in patients receiving chemotherapy carried a rs6815391 CT + TT genotype (95% *CI* 1.165 − 2.965), but there was no significant interaction between multiplication and addition. In the additive model, the risk of death in patients without chemotherapy carrying rs6815391 TT genotype was 2.062 times higher than that in patients receiving chemotherapy carrying rs6815391 CC genotype (95% *CI* 1.263 − 3.366), while there was no significant combined effect between rs6815391 additive subgroup and BMI subgroup. There was no significant multiplicative and additive interaction between rs6815391 additive, chemotherapy, and BMI subgroups (Table 5, Additional file 1).

There was no obvious combined effect between rs13409 and pulmonary disease, but there was a negative multiplicative interaction and antagonistic additive interaction. In the additive model, there was no obvious combined and multiplied interaction between rs13409 and lung disease, and the risk of death of patients with rs13409 TT genotype and family history of lung cancer was 3.234 times higher than that of patients with rs13409 CC genotype and no family history of cancer (95% *CI* 1.138 − 9.196). There was no significant additive interaction between rs13409 and a family history of lung disease and tumor (Table 6, Additional file 1).

## Discussion

In this study, clinical data related to the prognosis of lung cancer were collected, and the relationship between the four SNPs in the upstream *REX1* of *OCT4*, downstream *CTBP2* and *OCT4*, and overall survival of lung cancer prognosis was analyzed, but there was no significant correlation between each SNP locus and the overall survival time in various genetic models. Stratified analysis indicated that rs3740535 recessive AA genotype and additive GG genotype, rs3130932 dominant GT + GG genotype and additive TT genotype, rs13409 additive TT genotype, rs6815391 recessive CC genotype, and additional TT genotype were associated with an increased risk of lung cancer death. Rs3130932 recessive GG genotype was associated with a reduced risk of lung cancer death. Interaction based on stratified results exhibited that lung cancer patients with the GT + GG genotype in the dominant model of rs3130932 had an increased risk of death after blood metastasis, while patients with GG genotype in the recessive model had a reduced risk of death after radiotherapy or without blood metastasis. Patients carrying the TT genotype in the dominant model of rs6815391 with other pathological types had a reduced risk of death, while patients carrying CC genotype in the recessive model and TT genotype in the additional model had a significantly increased risk of death in patients not receiving chemotherapy. In the rs13409 additive model, TT genotype carriers with a family history of lung cancer had an increased risk of death.

Although the SNP loci were not statistically associated with the overall survival of those lung cancer patients in various genetic models, there was a statistically significant difference between the stratified subgroups. This indicates that cancer stem cells are heterogeneous among populations [22] or that different genetic factors contribute to the variations. In this study, we found that rs313932 in the *OCT4* gene, rs13409 in the *REX1* gene, and rs6815391 in the *CTBP2* gene interacted with some clinical features, suggesting that rs3130932, rs13409, and rs6815391 may be not independent risk factors for primary lung cancer. Transcriptional factors in embryonic stem cells interact to form networks that jointly regulate the fate of embryonic stem cells. *OCT4*, *NANOG*, Kruppel-like factor 4 (*KLF4*), SRY-related high mobility group Box2 (*SOX2*), and LIN28 are the basis for regulating stem cell pluripotency [23]. The *REX1* is a necessary factor for pluripotency and reprogramming, can replace *KLF4*, and is a new central actor controlling pluripotency [17]. Moreover, the *REX1* promoter contains the binding sites of multiple core transcription factors, which have a bidirectional regulatory effect on the *OCT4* gene and have an effect on the pluripotency of stem cells. *REX1* drives entry and exit pluripotency by lowering the reprogramming barrier (growth stagnation and apoptosis), facilitating mitochondrial fission, and transforming glycolytic metabolism from oxidative phosphorylation to glycolytic metabolism, dependent on the cyclin B1/ B2-DRP1 pathway, altering cell cycle progression and metabolic states. Actually, the *REX1* can regulate F9 cell differentiation by inhibiting the JAK/STAT (Janus kinase /signal and activator of transcription) signaling [24]. This signaling pathway is crucial for controlling the self-renewal of ES cells.

Downstream of *OCT4* regulation, it can also bind to related inhibitory complexes, the most prominent of which is found to be the nucleosome remodeling and deacetylation (NuRD) or histone deacetylase complex [25]. The C-terminal binding protein (*CTBP*) is an important ligand for its transcriptional inhibition function, and NuRD binds to *CTBP2* can regulate the NuRD-mediated deacetylation, facilitating embryonic stem cells to exit from pluripotency during differentiation [26]. *CTBP2* can also act on NuRD-related Bhara-like4 (*Sall4*) genes. The effect is amplified by binding to the TGF-β and Wnt signaling by ubiquitin-specific protease 9x (*USP9x*) and CXXC finger protein 5 (*CXXC5*) [27]. *CTBP* expression is upregulated in malignant tissues, and *CTBP2* is highly expressed in prostate cancer [28], breast cancer [29], esophageal cancer [30], liver cancer [31], and other common tumors. *CTBP2* can enhance the invasion and migration of cancer cells, and through some signaling pathways, it can regulate the cell cycle, and apoptosis, associated with tumor suppression [32, 33]. In addition, some SNPs affect gene function by modulating the number or structure of gene expression products, altering patients’ disease susceptibility, chemotherapy sensitivity, adverse reactions, and prognosis. Previous studies have shown that 0–6-methylguanine-DNA methyltransferase (O-6-methylguanine-DNA methyltransferase, *MGMT* gene, C2 (ATP Binding cassette subfamily C member 2, *ABCC2*), ATP-binding cassette transporter family class C4 (*ABCC4*), cytokine and inflammatory gene interleukin-6 (*IL-6*), prostaglandin-endoperoxide synthase 2 (*PTGS2*), and lymphotoxin-alpha (LTA), and multiple SNPs in the genes for events in the P38 mitogen-activated protein kinase (*P38 MAPK*) signaling are associated with different domains of lung cancer prognosis, with some genotypes having low quality of life and poor treatment prognosis [34–37]. In addition, a study of 400 lung cancer patients found that the glutathione peroxidase 7 (*GPX-7*) gene and *ABCC4* SNPs were associated with peripheral neuropathy following chemotherapy [38], attributing to the effect of SNP on gene expression.

In this study, we used hierarchical and multi-factor analyses of the data to effectively control the interference of confounding factors. To further explore the interaction between SNPs and related influencing factors, the multiple observations and multiple factors might enrich conclusions. We recognized the limitations of this study. First, given that the subjects were only from three hospitals in Fujian province, there might be some degrees of selection bias. It is difficult to extrapolate the results from other ethnic groups, because allele frequency patterns vary widely among ethnic groups. Secondly, this study only investigated the influencing factors of cancer stem cell-related SNPs on the prognosis of primary lung cancer. We neither have further experimental verification, nor determine the influencing mechanisms. Finally, because the prognosis is affected by a variety of environmental and genetic factors, the effect of a single SNP on quality of life may be limited, and haplotype studies based on SNPs may be more significant.

Although some progress has been made in lung cancer research worldwide, there are still many deficiencies in reducing the postoperative recurrence and metastasis rates to increase the 5-year survival rate of lung cancer. The results of this study may lay a foundation for further research after being verified in multi-center, rigorously designed large sample studies, and the specific mechanisms need to be further explored by relevant functional studies.

## Conclusions

Rs3740535, rs3130932, rs13409, and rs6815391 were associated with the prognosis of lung cancer patients, and their specific genotypes may affect the prognosis and survival time of lung cancer patients. Potentially, these findings may provide a functional basis for investigating the roles of the *OCT4*, *REX1*, and *CTBP2* genes in the development and progression of primary lung cancer. Rs3740535, rs3130932, rs13409, and rs6815391 alone or their polymorphic combinations may be promising prognostic biomarkers for primary lung cancer. However, further studies are warranted in different ethnic groups to validate the association between genetic polymorphisms of *OCT4*, *REX1*, and *CTBP2* genes and the overall survival of primary lung cancer to reveal the underlying molecular mechanisms.

### Supplementary Information

**Additional file 1.**

## Data Availability

The datasets used or analyzed during the current study are available from the corresponding author on reasonable request.
