# Supplementary material for: Relationship between cancer stem cell-related SNPs and survival outcomes in patients with primary lung cancer
Source: World J Surg Oncol. 2023 Aug 11;21:243. doi: 10.1186/s12957-023-03064-z (PMC10416443; doi:10.1186/s12957-023-03064-z)
Supplement: Supplementary file 1 — Additional file 1. [file 12957_2023_3064_MOESM1_ESM.docx]

| Table 4 Analysis of interaction between rs3130932 genotype and hematogenous metastasis and radiotherapy | | | | | | | | | | | | | | |
| --- | --- | --- | --- | --- | --- | --- | --- | --- | --- | --- | --- | --- | --- | --- |
| Dominant model | | |  | Implicit model | | | | |  | [Additive](javascript:;) [model](javascript:;) | | | | |
| [Genotype](javascript:;) | [Hematogenous](javascript:;) [metastasis](javascript:;) | *αHR*(95%*CI*)^*^ |  | [Genotype](javascript:;) | [Radiation](javascript:;) [oncology](javascript:;) | *αHR*(95%*CI*)^*^ | [Hematogenous](javascript:;) [metastasis](javascript:;) | *αHR*(95%*CI*)^*^ |  | [Genotype](javascript:;) | [Hematogenous](javascript:;) [metastasis](javascript:;) | *αHR*(95%*CI*)^*^ | Sex | *αHR*(95%*CI*)^*^ |
| TT | Yes | 1 |  | GT+TT | Yes | 1 | Yes | 1 |  | GG | Yes | 1 | Male | 1 |
| TT | No | 1.013（0.763-1.346） |  | GT+TT | No | 1.074（0.874-1.320） | No | 0.843(0.671-1.059) |  | GG | No | 1.008（0.738-1.377） | Female | 0.703（0.476-1.037） |
| GT+GG | Yes | **1.362（1.021-1.818)** |  | GG | Yes | **0.536（0.298-0.962）** | Yes | 1.481（0.960-2.287） |  | TT | Yes | 1.675（1.048-2.677） | Male | 0.760（0.564-1.023） |
| GT+GG | No | 0.834(0.629-1.105) |  | GG | No | 1.147（0.842-1.563） | No | **0.650（0.464-0.911）** |  | TT | No | 0.706（0.477-1.044） | Female | 1.028（0.584-1.809） |
| Multiplication interaction |  | **0.604（0.429-0.851）** |  | Multiplication interaction |  | **1.194（1.010-1.412）** |  | **0.520（0.309-0.878）** |  | Multiplication interaction |  | **0.418（0.238-0.735）** |  | **1.925（1.067-3.475）** |
| Relative excess risk(RERI) |  | -0.569(-6.620-5.483) |  | Relative excess risk(RERI) |  | 0.276（-0.071-0.623） |  | -735866.8（-672800200-671328466） |  | Relative excess risk(RERI) |  | 94784.56(-143133241-143322810) |  | 0.082(-0.318-0.483) |
| Attributable risk percent(ARP) |  | -0.144(-2.030-1.741) |  | Attributable risk percent(ARP) |  | 4.094（-8.576-16.765） |  | -3.024(-10.010-3.962) |  | Attributable risk percent(ARP) |  | 0.020(-1.981-2.020) |  | 0.500(-2.601-3.601) |
| [Synergic index](javascript:;)(S) |  | 0.838(0.088-7.996) |  | [Synergic index](javascript:;)(S) |  | 0.771（0.595-1.000） |  | 0.246(0.044-1.410) |  | [Synergic index](javascript:;)(S) |  | 1.020(0.133-7.851) |  | 0.910(0.599-1.383) |
| Note: * The adjusting factors are gender, age, first diagnosis metastasis, pleural metastasis, lymphatic metastasis, hematologic metastasis, BMI, treatment or surgery, maximum tumor diameter, pulmonary disease, education, smoking history, and clinical stages (when adjusting factors are included, corresponding joint items are not included in adjustment) | | | | | | | | | | | | | | |

| Table 5 Analysis of interaction between rs6815391 genotypes and family history of tumor, chemotherapy and BMI | | | | | | | | | | | | | | | |
| --- | --- | --- | --- | --- | --- | --- | --- | --- | --- | --- | --- | --- | --- | --- | --- |
| Dominant model | | | | |  | Implicit model | | |  | [Additive](javascript:;) [model](javascript:;) | | | | | |
| [Genotype](javascript:;) | Family history of cancer | *αHR*(95%*CI*)^*^ | [pathological](javascript:;) [pattern](javascript:;) | *αHR*(95%*CI*)^*^ |  | [Genotype](javascript:;) | [chemotherapy](javascript:;) | *αHR*(95%*CI*)^*^ |  | [Genotype](javascript:;) | [chemotherapy](javascript:;) | *αHR*(95%*CI*)^*^ | [Genotype](javascript:;) | BMI group | *αHR*(95%*CI*)^*^ |
| TT | No | 1 | [adenocarcinoma](javascript:;) | 1 |  | CT+TT | Yes | 1 |  | CC | Yes | 1 | CC | 18.5-24 | 1 |
| TT | Other tumors | 1.039（0.730-1.477） | [squamous](javascript:;) [carcinoma](javascript:;) | 0.873  (0.666-1.145) |  | CT+TT | No | 0.870（0.683-1.107） |  | CC | No | 0.905（0.659-1.243） | CC | <18.5 | 0.927（0.619-1.387） |
| TT | Lung cancer | 1.503 （0.975-2.317） | Others | **0.707**  **(0.512-0.978)** |  | CC | Yes | 0.912（0.667-1.247） |  | TT | Yes | 0.936（0.672-1.302） | CC | >=24 | 0.883（0.677-1.151） |
| CT+CC | No | 1.152（0.967-1.371） | [adenocarcinoma](javascript:;) | 0.888  (0.716-1.101) |  | CC | No | **1.859（1.165-2.965）** |  | TT | No | **2.062（1.263-3.366）** | TT | 18.5-24 | 0.971（0.686-1.374） |
| CT+CC | Other tumors | 0.941（0.684-1.295） | [squamous](javascript:;) [carcinoma](javascript:;) | 1.054  (0.810-1.372) |  |  |  |  |  |  |  |  | TT | <18.5 | 1.817（0.826-3.998） |
| CT+CC | Lung cancer | 1.126（0.708-1.791） | Others | 1.157  (0.854-1.566) |  |  |  |  |  |  |  |  | TT | >=24 | 1.432（0.869-2.362） |
| Multiplication interaction |  | 0.997（0.912-1.089） |  | 1.031  (0.965-1.102 |  | Multiplication interaction |  | 1.051（0.862-1.282） |  | Multiplication interaction |  | 1.178（0.933-1.487） | Multiplication interaction |  | 1.671（0.892-3.128） |
| Relative excess risk(RERI) |  | 0.045  (-0.826-0.915) |  | -0.100  (-0.426-0.226) |  | Relative excess risk(RERI) |  | 0.889  (-0.952-2.730) |  | Relative excess risk(RERI) |  | 1.155  (-1.415-3.726) | Relative excess risk(RERI) |  | -0.323  (-1.097-0.451) |
| Attributable risk percent(ARP) |  | 0.031  (-0.556-0.619) |  | -0.191  (-0.786-0.404) |  | Attributable risk percent(ARP) |  | 0.701  (0.268-1.135) |  | Attributable risk percent(ARP) |  | 0.723  (0.336-1.110) | Attributable risk percent(ARP) |  | -0.446  (-1.638-0.746) |
| [Synergic index](javascript:;)(S) |  | 1.119  (0.144-8.667) |  | 1.266  (0.430-3.728) |  | [Synergic index](javascript:;)(S) |  | -0.430 |  | [Synergic index](javascript:;)(S) |  | -1.070 | [Synergic index](javascript:;)(S) |  | -5.870 |
| Note: * The adjusting factors are gender, age, first diagnosis metastasis, pleural metastasis, lymphatic metastasis, hematologic metastasis, BMI, treatment or surgery, maximum tumor diameter, pulmonary disease, education, smoking history, and clinical stages (when adjusting factors are included, corresponding joint items are not included in adjustment) | | | | | | | | | | | | | | | |

| Table 6 Analysis of interaction between rs13409 genotypes and family history of pulmonary disease and tumor | | | | | | | | | |
| --- | --- | --- | --- | --- | --- | --- | --- | --- | --- |
| Dominant model | | |  | [Additive](javascript:;) [model](javascript:;) | | | | | |
| [Genotype](javascript:;) | [pulmonary](javascript:;) [disease](javascript:;) | *aHR*(95%*CI*)^*^ |  | [Genotype](javascript:;) | [pulmonary](javascript:;) [disease](javascript:;) | *aHR*(95%*CI*)^*^ | [Genotype](javascript:;) | Family history of cancer | *aHR*(95%*CI*)^*^ |
| CC | Yes | 1 |  | CC | Yes | 1 | CC | No | 1 |
| CC | No | 1.081（0.859-1.362） |  | CC | No | 1.136(0.892-1.446) | CC | Other tumors | 1.093（0.791-1.511） |
| CT+TT | Yes | 1.143（0.890-1.468） |  | TT | Yes | 1.048(0.684-1.605) | CC | Lung cancer | 1.062（0.659-1.711） |
| CT+TT | No | 0.891(0.709-1.120) |  | TT | No | 0.912(0.644-1.292) | TT | No | 0.900（0.680-1.191） |
|  |  |  |  |  |  |  | TT | Other tumors | 0.602（0.280-1.295） |
|  |  |  |  |  |  |  | TT | Lung cancer | **3.234（1.138-9.196）** |
| Multiplication interaction |  | **0.721（0.524-0.992）** |  | Multiplication interaction |  | 0.766（0.448-1.311） | Multiplication interaction |  | 1.073（0.914-1.258） |
| Relative excess risk(RERI) |  | **-0.583(-1.103-(-0.062))** |  | Relative excess risk(RERI) |  | -0.476  (-1.155-0.204) | Relative excess risk(RERI) |  | -0.082  (-0.605-0.440) |
| Attributable risk percent(ARP) |  | **-0.572(-1.711-0.568)** |  | Attributable risk percent(ARP) |  | -0.937  (-2.554-0.680) | Attributable risk percent(ARP) |  | -0.332  (-2.133-1.469) |
| [Synergic index](javascript:;)(S) |  | **0.033(4.055964e-36-2.653439e+32)** |  | [Synergic index](javascript:;)(S) |  | 30.224  (7.222735e-37-1.264739e+39) | [Synergic index](javascript:;)(S) |  | 1.123(0.492-2.563) |
| Note: * The adjusting factors are gender, age, first diagnosis metastasis, pleural metastasis, lymphatic metastasis, hematologic metastasis, BMI, treatment or surgery, maximum tumor diameter, pulmonary disease, education, smoking history, and clinical stages (when adjusting factors are included, corresponding joint items are not included in adjustment) | | | | | | | | | |
